# Macro- and Microelemental Composition and Toxicity of Unsweetened Natural Cocoa Powder in Sprague-Dawley Rats

**DOI:** 10.1155/2016/4783829

**Published:** 2016-08-17

**Authors:** Isaac Julius Asiedu-Gyekye, Samuel Frimpong-Manso, Benoit Banga N'guessan, Mahmood Abdulai Seidu, Paul Osei-Prempeh, Daniel Kwaku Boamah

**Affiliations:** ^1^Department of Pharmacology and Toxicology, University of Ghana School of Pharmacy, College of Health Sciences, Legon, Ghana; ^2^Department of Pharmaceutical Chemistry, University of Ghana School of Pharmacy, College of Health Sciences, Legon, Ghana; ^3^Department of Medical Laboratory Sciences (Pathology), School of Biomedical and Allied Health Sciences, University of Ghana, Legon, Ghana; ^4^Geological Survey Department, Accra, Ghana

## Abstract

Unsweetened natural cocoa powder (UNCP) is a pulverized high-grade powder of compressed solid blocks which remains after extraction. Little scientific data is available concerning its safety despite the presence of potential toxic elements. Elemental composition in UNCP was analyzed with ED-XRF spectroscopy. Single oral high dose toxicity study was conducted on adult male Sprague-Dawley rats (150 g) by the limit test method. One group received water and the test group 2000 mg/kg UNCP. All animals were observed for 14 days and then euthanized for haematological, biochemical, and histopathological examinations. Thirty-eight (38) elements were found in UNCP. There was an increase in HDL cholesterol (*p* < 0.05), reduction in LDL cholesterol (*p* > 0.05), alkaline phosphatase (*p* < 0.05), and creatinine levels, and slight increase in urea levels (*p* > 0.05). Haematological changes were not significant. Histopathological analysis showed no toxic effect on the heart, liver, kidney, lungs, testis, and spleen. Intestinal erosion was observed in the test group. UNCP appears to be relatively safe when taken as a single oral high dose of 2000 mg/kg b.w.t. in rats. Caution should however be exercised at high doses due to the high elemental content of copper and high possibility of intestinal lining erosion.

## 1. Introduction

Natural products (secondary metabolites) including cocoa have been the most successful source of potential drug leads [1, 2]. Extensive research is being carried out on plant materials gathered from the rain forests and other places for their potential medicinal value and potential toxic effects [3]. As such, the demand for herbal remedies has been increasingly rising in industrialized countries as it is in developing countries [4].

The medicinal and pharmacological importance of* Theobroma Cacao* and its powder, unsweetened natural cocoa powder (UNCP) as nutraceutical and as traditional medicine, has been well investigated. In Ghana and other parts of Africa, UNCP is used as remedy for managing bronchial asthma, as an aphrodisiac, antidiabetic, anti-inflammatory, cardioprotective, antihypertensive, and antimalarial agent [5–11]. In Ghana and West Africa, UNCP is a common beverage [1].

Cocoa powder is prepared after removal of the cocoa butter from powdered cocoa beans via fermentation, drying and bagging, winnowing, roasting, grinding, and pressing. The solid blocks of compressed cocoa remaining after extraction (press cake) are pulverised into a fine powder to produce a high-grade cocoa powder.

The chemical composition of cocoa has been well investigated using various methods [12–15]. UNCP contains about 1.9% theobromine and 0.21% caffeine [16]. Proanthocyanidin being a constituent of unsweetened natural cocoa powder has been found to be capable of destroying the mucosal lining of the gastrointestinal tract [17]. Besides, micro- and macroelements present in plants may interfere with the availability of secondary metabolites in UNCP which may easily modulate their pharmacological activity [18]. The presence of toxic heavy metals in medicinal plants can also pose as a threat to the health of consumers [19]. For the safety of consumers, the World Health Organization states maximum permissible levels in raw plant materials for only cadmium (0.3 mg kg^−1^), arsenic (1 mg kg^−1^), and lead (10 mg kg^−1^) [20].

Toxicity studies on this nutraceutical are rare. Cocoa per se has not attracted much interest to the scientific world probably because of its long term usage with very little reported adverse effects. There have been reports on the potential carcinogenicity and teratogenicity, that is, bilateral testicular atrophy and aspermatogenesis of cocoa [21, 22]. Besides, Sertoli cells have been identified as the main target for theobromine toxicity accompanying cocoa administration [23].

It is against this background that this study is being conducted to determine the elemental composition and safety of this important nutraceutical.

## 2. Materials and Methods

### 2.1. Energy Dispersive X-Ray (ED-XRF) Measurements

Generally, the X-ray fluorescence (XRF) is a fast, accurate, and nondestructive analytical technique used for the elemental and chemical analysis of powdered, solid, and liquid samples [19, 24–26].

Sample of processed cocoa powder was purchased (Batch number BT620IT; FDA/DK06-070) from a supermarket. The sample was sieved using sieve of 180 microns. Three samples were prepared and sieved with a mesh size (aperture) of 180 *μ*m into a fine powder and kept in a dry well-labelled container. Before pelletation, the sample was kept in an oven at 60°C overnight. Due to their morphology and the loose nature, triplicate weighed samples—4000 mg/sample—were added separately to 900 mg Fluxana H Elektronic BM-0002-1 (Licowax C micropowder PM-Hoechstwax) as binder; the mixture was homogenized using the RETSCH Mixer Mill (MM301) for 3 min and pressed manually with SPECAC hydraulic press for 2 min with a maximum pressure limit of 15 tons (15000 kg) into pellets of 32 mm in diameter and 3 mm thickness for subsequent XRF measurements. Time between pelletation and measurement was kept short to avoid deformation of the flat surfaces of the pellets. SPECTRO X-Lab 2000 spectrometer (Geological Survey Department, Accra, Ghana) enhanced with three-axial geometry to reduce background noise due to radiation polarization and its monochromatic radiations emitted from the X-ray tube to excite the atoms of the samples were used for simultaneous analysis and measurement of the elemental content of the samples. This spectrometer is equipped with Rh anode and 400 W Pd X-ray tube, a 0.5 mm Be end window tube, a Si (Li) detector (resolution of 148 eV – 1000 cps Mn K*α*), available targets (Al_2_O_3_ and B4C used as a BARKLA polarizer), HOPG (High Oriented Pyrolitic Graphite) as a BRAGG polarizer, Al, Mo, and Co as secondary target, and 0.5 mm Be side window. It has a carousel (circular rotating sample changer) inside a sample chamber with a capacity of 20 sample holder disc (32 mm) for sequential sample analyses. The radiation chamber was cooled using liquid nitrogen. Its computer-based multichannel analyzer-SPECTRO X-Lab Pro Software package (Turbiquant) controlled and computed spectral analysis and collected, evaluated, and stored data. Combination of these different targets gave a typical detection limit for light elements (Si, Al, Mg, and Na) in the range of 25–50 ppm. For heavy metals, 1–5 ppm was the limit of detection. This spectrometer was factory calibrated using a number of international rock standards.

### 2.2. Preparation of UNCP

Calculated amount (9.6 g) of Brown Gold Natural Cocoa Powder from Hords Company Ltd., (*Batch number BT620IT*) registered with the Ghana Food and Drugs Authority (*FDA/DK06-070*) was dissolved in warm distilled water (40 mL) with stirring making a concentration of 240 mg/mL (of the UNCP). The preparation was then administered to the animals via oral gavage based on their individual body weights.

### 2.3. Animal Experimentation

Twenty (20) adult male Sprague-Dawley rats of average weight 150 g were purchased from the Animal House Department of the Korle-Bu Teaching Hospital, Korle-Bu, Accra. The rats were acclimatized to laboratory environment (20–24°C), 60 ± 1% humidity with a 12 h light-darkness cycle for 7 days prior to experimentation. The rats had access to standard laboratory diet and water* ad libitum.* The experimental procedures were approved by the departmental ethical and protocol review committee and the Noguchi Memorial Institute for Medical Research Institutional Animal Care and Use Committee with protocol number 2013-01-3E and also conducted in accordance with international ethical guidelines.

### 2.4. Experimental Design

The Sprague-Dawley rats were randomly assigned to the experimental group and the control group for 7 days before the start of the experiment. Both groups contained ten (10) rats each. All rats had access to water and food except for a 12 hour fasting period before the administration of the unsweetened natural cocoa powder. The experimental group of rats received the unsweetened natural cocoa powder at the dose of 2000 mg/kg while the control group received an equal volume of distilled water. This was based on the fact that the initial testing of 300 mg/kg and 1200 mg/kg to single rats each did not result in any death.

### 2.5. Effect of UNCP on Body and Relative Organ Weights

Selected organs like the liver, kidney, heart, lungs, testis, spleen, and intestines were excised quickly and placed in ice-cold saline to wash off blood, trimmed of fat and connective tissues, blot dried, and finally weighed on a balance. The organ-to-body weight index (OBI) was calculated as the ratio of organ weight and the body weight of the animal before sacrifice ×100. Body weight of rats were also taken dosing, a week after dosing on Day 7 and before sacrificing them on Day 14.

### 2.6. Effect of UNCP on Haematological Parameters

Two millilitres (2 mL) of blood from euthanized SD rats was drawn out by cardiac puncture and then transferred into EDTA test tubes. An automated haematology analyzer was used to estimate the counts of the various parameters considered in this study. Peripheral blood smear was also done to examine the nature of blood cells.

### 2.7. Effect of UNCP on Serum Biochemistry

1 mL of the blood of sacrificed rats was collected by means of cardiac puncture. The blood sample was allowed to stand and then centrifuged at 4000 rpm for 15 minutes using a Wiperfuge centrifuge with the serum collected separately into Eppendoff tubes for the measurement of the biochemical parameters.

### 2.8. Histopathology Examination

The liver, kidney, heart, lungs, spleen, testis, and small intestinal organs were immediately fixed in 10% buffered formaldehyde solution for 24 h. Samples of the tissues were then paraffin embedded and sectioned at 5 *μ*m thickness. Sectioned tissues were mounted on slides and stained with haematoxylin and eosin (H&E). The sections were evaluated microscopically for histological changes under a light microscope (Olympus BX 51TF).

### 2.9. Statistical Analysis and Data Evaluation

Statistical analysis of the data was done using GraphPad Prism Software version 5.0. Results were expressed as mean ± standard error of mean, *n* = 5. Significant difference between dosed groups and control was evaluated by performing student's one-tailed *t*-test. *p* values less than 0.05 were considered statistically significant.

## 3. Results

### 3.1. Energy Dispersive X-Ray (ED-XRF) Measurements

A total of thirty-eight (38) macro-12 elements (sodium (Na), magnesium (Mg), aluminium (Al), silicon (Si), phosphorus (P), sulphur (S), chlorine (Cl), potassium (K), calcium (Ca), titanium (Ti), manganese (Mn), and iron (Fe)) and micro-26 elements (vanadium (V), chromium (Cr), cobalt (Co), nickel (Ni), copper (Cu), zinc (Zn), gallium (Ga), arsenic (As), rubidium (Rb), strontium (Sr), yttrium (Y), zirconium (Zr), niobium (Nb), molybdenum (Mo), antimony (Sb), iodine (I), cesium (Cs), barium (Ba), lanthanum (La), cerium (Ce), hafnium (Hf), tantalum (Ta), lead (Pb), bismuth (Bi), thorium (Th), uranium (U)) (Table 1) were identified and evaluated.

### 3.2. Microelements

These elements either in % w/w or in ppm were converted to their respective amounts in milligrams. For example, an average of triplicate measurements of elements such as magnesium (Mg) in percentage was converted as (0.837 + 0.83 + 0.809/3 = 0.8253/100*∗*4000 mg = 33.0133 mg 4000 mg^−1^) and lead (Pb) in parts per million (ppm) was calculated as an average of triplicate measurement:

(1) 0.9+0.9+0.930.91000000∗4000=0.0036 mg  4000 mg−1.

Simple statistics (mean and standard) of the results were calculated to gain a better understanding of the results (Table 1).

### 3.3. Effects of Treatment on Food, Water Intake, and Body Weight

Food and water intake of the treated animals that received 2000 mg/kg body weight and control group remained relatively the same. There was generally no increase in the intake of food and water by both the control and experimental SD rats. General physical observations such as abnormality of the eyes, skin and fur, coma, convulsion, tremors, diarrhea, lethargy, sleep, morbidity, and then mortality were all not observed to have happened.

From Day 1 to Day 14, there were no significant changes in the body weight of the SD rats that survived at the end of the experiment as in Table 3. However, there was a slight decrease in the weight of the treatment group from Day 1 to Day 14.

### 3.4. Clinical Signs and Mortality Patterns

At the dose level tested, no untoward clinical signs were observed in all the rats used. There were no changes in the nature of the stool, urine, and an eye colour of all the rats. No mortality was observed in the treatment or control rat groups.

### 3.5. Effects on Relative Organ Weight

There were no significant changes in the relative weights of the liver, kidney, heart, lungs, spleen, and testis of the treated rats in relation to the control groups. However, the treated rats consistently showed slightly reduced organ weight values as compared to the control group rats. This is as shown in Table 3.

### 3.6. Effects on Haematological Parameters

There were generally no significant changes in the various haematological parameters of the treatment group in comparison with the control group as shown in Table 3. There was an increased value for the percentage eosinophil and monocyte of the treated group as compared to the control group. This increase was however not statistically significant. Similarly there was a marginal increase for the eosinophil and basophil numbers of the treatment group in comparison with the control group. The platelet number for the treatment groups however reduced in comparison with that of the control group.

The peripheral blood smear also showed no significant changes in the size, colour, and nature of the red blood cells and white blood cells when the treatment group was compared with the control group.

### 3.7. Effects on Serum Biochemistry

Biochemical profile which includes liver function indices, kidney function indices, and lipid profile of the treated rats and that of the control rats are presented in Table 4.

The acute oral administration of UNCP at the limit dose of 2000 mg/kg body weight did not cause any statistically significant change in the serum proteins and bilirubin as well as some electrolytes (slight decrease in sodium and slight increase in potassium). Blood urea nitrogen increased (*p* > 0.05) while creatinine levels reduced (*p* > 0.05) compared to the control. The levels of the liver marker enzymes (aspartate transaminase, gamma glutamyl transferase, and alanine transferase) of treated animals were not significantly different from that of the control. There was however a reduction in ALP levels (*p* < 0.05). Changes in serum biochemistry in male SD rats receiving 2000 mg/kg b.w.t. of UNCP are shown in Table 5 and changes in haematological indices in male SD rats receiving 2000 mg/kg b.w.t. of UNCP are shown in Table 6.

### 3.8. Histopathological Changes

#### 3.8.1. Effects of UNCP Treatment on Histology of the Liver, Kidney, Heart, Lungs, Testis, Spleen, and Small Intestines

**Figure 1:**
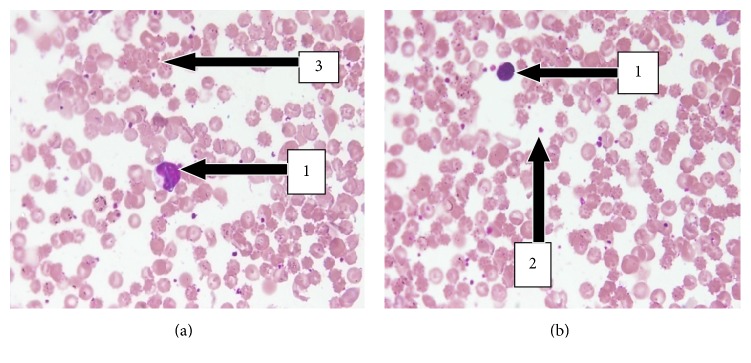
Leishman stained peripheral blood smear plate (20x). (a) Peripheral blood smear plate of control male SD rats showing normal distribution of blood cells. Note the white blood cell (1), platelet (2), and red blood cell (3). (b) Peripheral blood smear plate of treated animals of dose 2000 mg/kg showing normal distribution of WBCs, RBCs, and platelets. Note the normocytic and normochromic nature of the RBCs.

**Figure 2 fig2:**
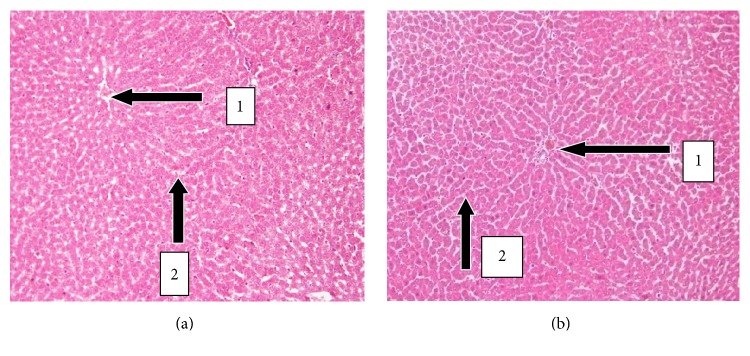
H&E stained section of livers at 20x magnification. (a) Liver sections of control rat showing normal histology. Note the presence of the central vein with no congestion and cellular infiltration (1) and sinusoids with no dilatation (2). (b) Liver sections of treated male SD rats (2000 mg/kg) with normal central vein, sinusoids, and hepatocytes.

**Figure 3 fig3:**
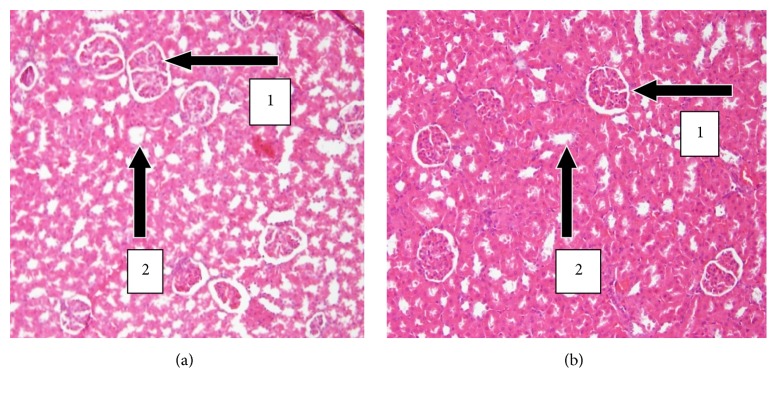
H&E stained sections of kidneys at 20x magnification. (a) Kidney sections of control male SD rats showing normal histology. Note the presence of the glomerular capsule with no necrosis, degeneration, and congestion (1) and normal convoluted tubules with no tubular casts (2). (b) Kidney section of treated animals (2000 mg/kg) with normal glomerulus and renal tubules with no congestion and cellular infiltration.

**Figure 4 fig4:**
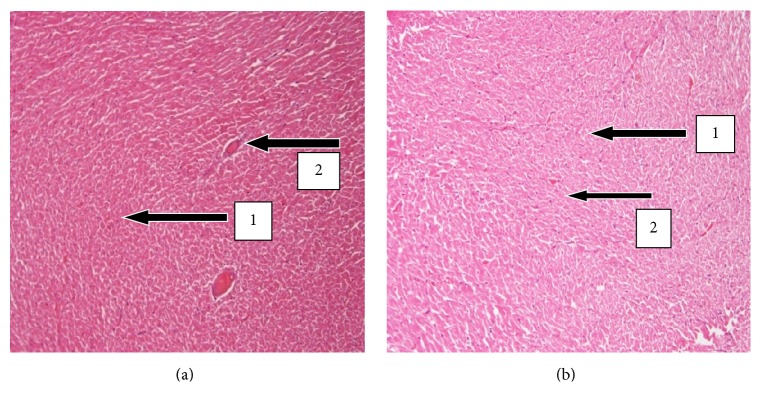
H&E stained sections of hearts at 20x magnification. (a) Heart sections of control SD rats showing normal myocardium. Note the presence of the myocardial fibres with no necrosis (1) and coronary vessels carrying blood normally to the heart (2). (b) Heart sections of treated animals (2000 mg/kg) showing normal histology and branched myofibrils.

**Figure 5 fig5:**
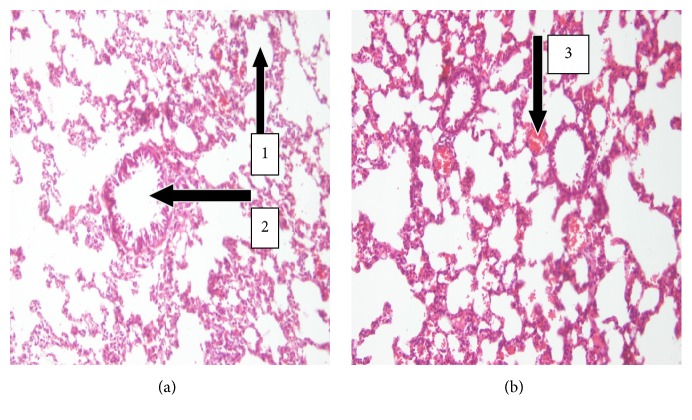
H&E stained sections of lungs at 20x magnification. (a) Lung sections of control rats showing normal histology. Note the presence of the alveoli with its surrounding interalveolar septa (1), bronchus (2), and pulmonary vessels with normal blood flow (3). (b) Lung sections of treated animal (2000 mg/kg) showing normal structure of the bronchi and alveoli.

**Figure 6 fig6:**
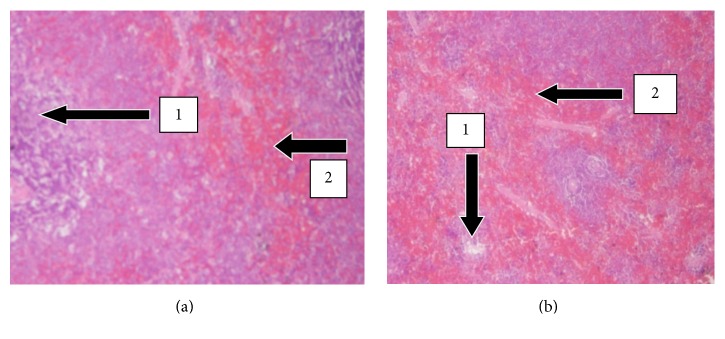
H&E stained sections of the spleen at 20x magnification. (a) Spleen sections of control SD rats showing normal histology. Note the presence of the white pulp (1) and the red pulp (2). (b) Spleen sections of treated male SD rats (2000 mg/kg) of UNCP showing intact splenic pulps.

**Figure 7:**
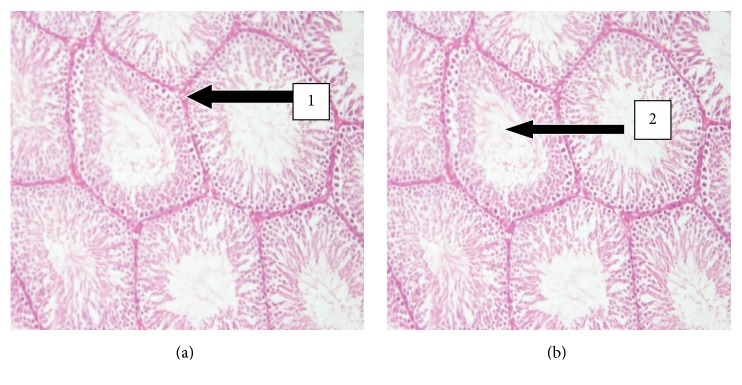
H&E stained sections of the testis at the magnification of 20x. (a) Testis sections of control SD rats showing normal histology. Note the presence of the intact seminiferous tubules (1) and the Sertoli cells which produces spermatozoa (2). (b) Testis sections of treated animals (2000 mg/kg) showing normal seminiferous tubules with intact spermatogenesis.

The results of histopathology changes in the liver, kidney, heart, lungs, testis, spleen, and small intestines are summarized in Figures 1 [Fig fig2] [Fig fig3] [Fig fig4] [Fig fig5] [Fig fig6]–7. The various organs from the control group had a normal histology and appearance. Generally, there were no observable changes in the architecture of the various organs of the treatment rats in comparison with the control.

The histology of the liver and the kidney was consistent with the normal liver and kidney function indices obtained in the serum biochemical analysis. The lipid profile was also consistent with the normal nature of the heart and the liver since both organs had no fatty tissues on observation.

However, the histology of the small intestines of animals that received UNCP showed mild changes as compared to that of the control rats. There were mild erosion of the lining of the small intestines. As such there was only a mild inflammatory response with less cellular infiltration.

## 4. Discussion

Most people believe that herbal medicines have no side effects or any potential risks due to their natural origins and are often considered as healthy food supplements and not drugs. Most herbs used for medicinal purposes are usually prescribed by the consumers and there is a lack of control and review concerning the dose, frequency, and route of administration. Active components found in these medicinal herbs have the potential of causing toxicity in humans.

This study focused on the acute toxicity effect of UNCP in male SD rats. The increased use of this natural product has called for concerns over both the efficacy and safety of the product.

UNCP contains phytochemicals such as tannins, saponins, cardiac glycosides, terpenoids, and flavonoids [27] which are normally responsible for both therapeutic and toxic effects of various plant and herbal extracts or products [28].

Evaluating UNCP as nutraceutical, the assumption was that the average African weighs 60.70 kg. UNCP will provide the following nutritional values per every 4000 mg of UNCP and its corresponding % RDA as represented in Tables 1 and 2. Magnesium contributes about 25% of the minimum amount of magnesium the human body requires per day (Table 2). Evaluating UNCP's medicinal value, for example, elements believed to be involved in the pathophysiology of hypertension and dysrhythmias and other cardiovascular diseases [29–31], sodium 0.51% (both men and women), magnesium 25% (men) and 32% (women), potassium 10%, and calcium 3%, were considered. High copper 104% (both men and women) and chromium (3750% in men/5250% in women) both implicated in pathophysiology of diabetes further justify cocoa's traditional usage as traditional medicine.

WHO's permissible limits of lead and arsenic are 0.00016 mg/kg and 0.0010 mg/kg, respectively [32]. Heavy metals determined in UNCP were Pb – 0.0036 mg and As – 0.002 mg corresponding to 0.0002 mg/kg and 0.0001 mg/kg, respectively, with the assumption that the average African weighs 60.7 kg. These values are far below WHO guidelines (Table 2). The high content of Cu^2+^ (0.2984 mg per 4 g UNCP) should be of concern especially at high doses since copper has been shown to play a role in the pathogenesis of Wilson's syndrome and liver damage [33, 34] while the high content of chromium could have beneficial effect in the management of diabetes mellitus and cardiovascular disorders [17, 29, 35]. The relationship between these elements, nutrition, and medicine observed suggests that micro- and macroelements of herbal products should not be envisage always as contaminants.

There are concerns however with regard to the copper content in UNCP; with inference from dose translation from animal to human studies according to Reagan-Shaw et al. [36] which takes into account the body surface area of the animal species and man, then *K*_*m*_ (i.e., body weight/surface area) for human adult and rat could be estimated as 37 and 6, respectively [36]. The human equivalent dose (HED) of 2000 mg/kg UNCP in rats corresponds to approximately 324.32 mg/kg HED. This is equivalent to 19,686.224 mg UNCP (approx. 8 teaspoonful if a teaspoonful of UNCP = 2.5 g) for a normal human weight of 60.70 kg. The amount of copper contained in 19,686.224 mg UNCP is 1.469 mg. This may imply that an individual weighing 60.70 kg could have detrimental consequences if 8 teaspoonfuls of UNCP are ingested especially equivalent amounts on a daily basis.

Body weight changes between the control group and the experimental group that were taken on Day 1, Day 7, and Day 14 with respect to dosing were found not to be statistically significant (*p* > 0.05). The increase in the body weight for both groups for the first week after dosing was about 10 grams (8.3%), which was consistent with other observations [37]. This could be attributed to the increase in the food consumption of the animals within the first week after dosing. There was however a decrease in weight of the SD rats on the 2nd week after dosing by 8.3% and 22.2% (*p* < 0.05) for the control and test group, respectively. The decrease in body weight with the control animals is consistent with the corresponding decrease in their food and water intake while that for the test animals might be explained by the reduction in the food and water intake as well as the ability of UNCP to react with nutrients in the body including stored fat, carbohydrate, and protein [6].

Generally there was a 10.90% (*p* > 0.05) reduction of the organ weight in the test group as compared to the control group. The relative organ weight of the control and experimental group of SD rats was however very similar and thus no significant changes in organ weight were observed.

Hepatic assessment revealed a significant decrease in ALP (*p* < 0.05) and a slight reduction in bilirubin levels (*p* > 0.05) of the UNCP treated group in comparison with the controls. AST and ALT levels were not much affected (*p* > 0.05) by the administration of UNCP solution (Figure 5) as have been observed by other researchers [38, 39]. The plasma protein levels remained relatively unchanged compared with the controls (*p* > 0.05) which may indicate that UNCP has not got any adverse effect on the liver, a situation that is consistent with the histopathological results. It is most likely that the liver being an organ capable of regenerating damaged tissue may not be impaired early following an insult from a toxicant [40].

There was an increase in HDL cholesterol (*p* < 0.05), a decrease in the level of triglycerides, and LDL cholesterol of the UNCP group (*p* > 0.05) in comparison with that of the control while cholesterol levels remained relatively unchanged (Figure 5). This is consistent with the findings of Hammerstone et al. [41] that flavonoids and procyanidins [17] in UNCP possess lipid lowering abilities. The first human clinical study performed showed that 35 g of delipidated cocoa decreased LDL oxidation between 2 h and 4 h after ingestion [17]. An increase in HDL cholesterol (*p* < 0.05) and reduction in LDL cholesterol of the treated group in comparison with the control group were however recorded which agrees with the work of Galleano et al. [35]. This might possibly explain the antihyperlipidaemic and antihypertensive effect of UNCP observed in other studies [7, 10, 11]. The slight reduction in Na levels supports the diuretic effect of UNCP beneficial in blood pressure control. However, this urine output was not monitored neither did the test animals show significant increase in water consumption. It is highly possible that a much prolonged administration of UNCP could have had significant and pronounced effects on these parameters.

There was a slight increase in the level of urea and K^+^ (*p* > 0.05) while creatinine levels reduced in the UNCP group as compared to the control group (*p* > 0.05) (Figure 6). Histopathology evaluation of the liver, kidney, heart, lungs, spleen, and the testis of the animals that received UNCP showed no toxic effect as compared to that of the control group. It is obvious that both hepatic and renal effects which showed normal morphology in the treated male SD rats and control are consistent with the results for the kidney and liver function tests. UNCP solution therefore is not likely to have toxic effect on the kidney when administered in a single oral high dose of 2000 mg/kg.

Haematological results showed a decrease (28.44%, *p* > 0.05) in the level of platelet in the UNCP group in comparison with the controls. Polyphenols in cocoa have been found to reduce platelet count. Neutrophil and lymphocyte polymorph of white blood cells showed a slight decrease of 8.02% and 18.73%, respectively (*p* > 0.05) (Figure 6). Negligible difference between the experimental and control group was observed in the case of haemoglobin (1.48%) and red blood cells (2.77%, *p* > 0.05). These results are contrary to other studies where a forty-eight day administration of an aqueous Venaco cocoa powder was found to increase platelet and white blood cell levels while liver enzymes, HCT, and HGB levels remained relatively unchanged [42]. However, there have been other studies with unexplainable contradictory results where platelets and leucocytes have increased during natural cocoa administration [43, 44]. There remains much investigation into these scientific observations. Besides, there was no significant difference between the actual measurement of the width of the erythrocyte distribution curve and the mean erythrocyte size (RDW-CV and RDW-SD) when compared to the control (*p* > 0.05). This is confirmed by the peripheral blood smear which showed normal red blood cells with respect to their shape, size, and colour (Figure 1). They were therefore normocytic and normochromic. Thus UNCP is likely not to have produced any toxic effect on the red blood cell, white blood cell, and platelet according to the peripheral blood smear.

**Figure 8:**
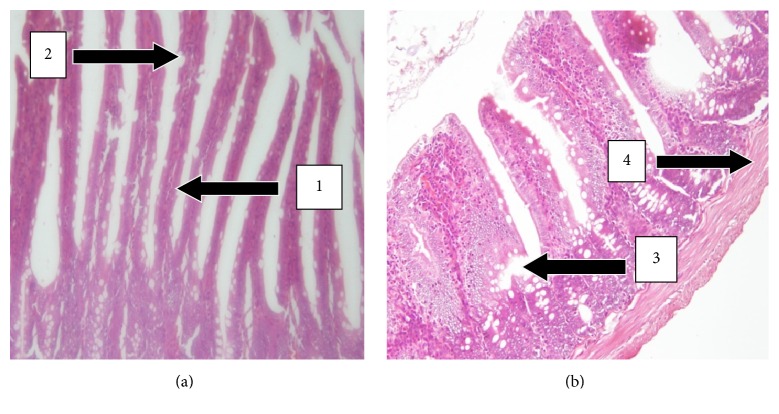
H&E stained sections of the small intestines at 20x magnification. (a) Small intestine sections of control male SD rats showing normal histology. Note the presence of the villi (1), goblet cells which produces mucus (2), and basement membrane with no proliferation (4). (b) Small intestine section of the UNCP treated male SD rats (2000 mg/kg) showing moderate changes and erosion of the mucosal lining of the villi (3).

One notable effect of UNCP was on the small intestines in the form of erosions of the mucosal lining of the villi, an effect which was not observed in the animals that received equivalent volumes of the vehicle (distilled water) (Figure 8). This could be caused by the high concentration of proanthocyanidins contained in the 2000 mg/kg dose of UNCP (approx. 2.5 g in man). This proanthocyanidins have been found to instigate the destruction of the mucosal lining of the gastrointestinal tract, haemorrhagic gastroenteritis in rabbits, striking lesions in the digestive tract of sheep, and congestion of the intestinal wall in rats [17]. Though considered safe, proanthocyanidin-rich products at high concentrations could result in intestinal erosions. Since the UNCP used in this study is a nonalkalized powder, it is likely to have a greater percentage of total polyphenols, increased epicatechin, and proanthocyanidins as compared to alkalized cocoa powder [45]. This should be a caution for individuals who regularly take high quantities of UNCP as beverage or those with peptic ulcer diseases, since UNCP has some level of cumulative properties.

The morphology of the spleen in the control and the UNCP group also showed normalcy with no tissue necrosis, hyperplasia, or depopulation and thus was concurrent with the results of the haematological analysis.

The lungs, testis, and heart also showed no signs of toxicity in the experimental animals and thus unsweetened natural cocoa powder could be considered to have no toxic effect on these organs at the dose administered in SD rats. In another study, Tarka et al. (1982; 1991) have shown the potential of cocoa to produce teratogenic and reproductive toxicity during chronic administration [21, 22]. This study has attempted to relate the effect of high dose nonalkalized UNCP and its elemental composition in laboratory animals.

## 5. Conclusion

In conclusion, the aqueous solution of unsweetened natural cocoa powder administered at the single oral high dose of 2000 mg/kg appears to be relatively safe in male SD rats. Caution should however be taken when using UNCP especially in high quantities or amounts since it is capable of causing considerable damage to the mucosal lining of the small intestines.

## Figures and Tables

**Table 1:** Mean and standard deviation (SD) of measured elements (mg/4000 mg).

	Element	Mean/SD mg/4000 mg
Macroelements	Na	2.4666 ± 0.00
	Mg	33.0133 ± 0.02
	Al	14.0093 ± 0.01
	Si	15.3880 ± 0.02
	P	64.3866 ± 0.00
	S	30.9120 ± 0.00
	Cl	2.3616 ± 0.00
	K	149.0667 ± 0.03
	Ca	11.0146 ± 0.00
	Ti	0.0232 ± 0.00
	Mn	0.4093 ± 0.00
	Fe	1.0309 ± 0.00

Microelements	V	0.2320 ± 1.73
	Cr	0.4200 ± 17.44
	Co	0.0108 ± 0.10
	Ni	0.0638 ± 1.16
	Cu	0.2984 ± 1.71
	Zn	0.4086 ± 0.74
	Ga	0.0024 ± 0.00
	As	0.0020 ± 0.00
	Rb	0.1698 ± 0.49
	Sr	0.1064 ± 0.20
	Y	0.0016 ± 0.00
	Zr	0.0125 ± 0.42
	Nb	0.0070 ± 0.29
	Mo	0.0044 ± 0.00
	Sb	0.0043 ± 0.06
	I	0.0133 ± 0.15
	Cs	0.0232 ± 0.10
	Ba	0.0620 ± 5.81
	La	0.0480 ± 0.00
	Ce	0.0849 ± 4.97
	Hf	0.0148 ± 0.17
	Ta	0.0213 ± 0.06
	Pb	0.0036 ± 0.00
	Bi	0.0024 ± 0.00
	Th	0.0020 ± 0.00
	U	0.0112 ± 0.10

**Table 2:** Recommended daily allowance: mg - (RDA, lit values) and percentage RDA of elements in UNCP.

Element	RDA (men)	RDA (women)	% RDA (men)	% RDA (women)
Na	1500 mg	1500 mg	0.51	0.51
Mg	420 mg	320 mg	24.60	32.30
P	700 mg	700 mg	28.70	28.70
Cl	2300 mg	2300 mg	0.32	0.32
K	4700 mg	4700 mg	10.00	10.00
Ca	1000 mg	1000 mg	3.40	3.40
Mn	2.3 mg	1.8 mg	56.50	72.20
Fe	8 mg	18 mg	40.25	17.90
Cr	35 *µ*g	25 *µ*g	3750.00	5250.00
Cu	900 *µ*g	900 *µ*g	103.60	103.60
Zn	11 mg	8 mg	11.60	16.00
Mo	45 *µ*g	45 *µ*g	30.60	30.60
I	150 *µ*g	150 *µ*g	27.78	27.78

**Table 3:** Changes of body weight of adult male SD rats treated with 2000 mg/kg body weight of solution of UNCP.

DAY	CTRL	UNCP	*p* value
Day 1	112.5 ± 12.50	142 ± 8.000	0.0526
Day 7	115.0 ± 10.00	142.0 ± 7.176	0.3618
Day 14	112.5 ± 7.500	135.0 ± 7.000	0.0522

**Table 4:** Changes in relative organ weight of male SD rats dosed with 2000 mg/kg body weight of UNCP solution.

ORGANS	CTRL	UNCP	*p* value
Liver	8.605 ± 4.225	7.454 ± 1.263	0.3260
Kidney	0.6000 ± 0.05000	0.5500 ± 0.02739	0.3618
Heart	8.605 ± 4.225	7.454 ± 1.263	0.3534
Lungs	0.8500 ± 0.05000	0.7600 ± 0.02915	0.3618
Spleen		7.454 ± 1.263	0.0829
Testis		1.224 ± 0.02502	0.3618

**Table 5:** Changes in serum biochemistry in male SD rats receiving 2000 mg/kg b.w.t. of UNCP.

Parameter	UNITS	CTRL	UNCP	*p* value
Creatinine	*µ*mol/L	43.25 ± 2.925	40.61 ± 1.158	0.3618
Urea UV	mmol/L	8.605 ± 4.225	8.854 ± 1.263	0.2880
Bilirubin total	*µ*mol/L	0.795 ± 0.0144	0.625 ± 0.165	0.3960
ALT	U/L	125 ± 0.722	120 ± 5.01	0.1999
Albumin	g/L	40.5 ± 0.442	39.0 ± 0.692	0.2046
AST	U/L	2.49 ± 0.358	2.52 ± 0.541	0.3263
Total protein	g/L	73.1 ± 1.02	70.6 ± 3.09	0.6061
Triglycerides	mmol/L	1.28 ± 0.160	0.943 ± 0.116	0.1185
Bilirubin direct	*µ*mol/L	0.555 ± 0.0240	1.38 ± 0.489	0.1791
ALP	U/L	707 ± 7.86	535 ± 40.7	0.0103
GGT	U/L	1.20 ± 0.200	2.40 ± 1.44	0.4316
HDL cholesterol	mmol/L	0.560 ± 0.0372	0.755 ± 0.0349	0.0060
Cholesterol	mmol/L	2.08 ± 0.0854	2.15 ± 0.129	0.6616
LDL cholesterol	mmol/L	1.27 ± 0.0740	0.934 ± 0.124	0.0810
Na^+^	mmol/L	137 ± 0.479	133 ± 0.477	0.0002
K^+^	mmol/L	5.75 ± 0.132	6.44 ± 0.293	0.1098
Ca^2+^	mmol/L	0.845 ± 0.0132	0.858 ± 0.0180	0.5200

**Table 6:** Changes in haematological indices in male SD rats receiving 2000 mg/kg b.w.t. of UNCP.

Parameter	Ctrl	UNCP	*p* value
WBC	8.605 ± 4.225	7.454 ± 1.263	0.3618
Neut. number	2.020 ± 0.4400	1.858 ± 0.3836	0.4109
Lymph number	5.985 ± 3.435	4.864 ± 1.111	0.3422
Mono. number	0.3700 ± 0.220	0.3740 ± 0.1225	0.4936
Eosin. number	0.2250 ± 0.125	0.3520 ± 0.07439	0.2045
Baso. number	0.0050 ± 0.005	0.0060 ± 0.002449	0.4228
Neut.%	27.65 ± 8.450	25.38 ± 4.450	0.4021
Lymph.%	65.80 ± 7.600	63.90 ± 6.119	0.4348
Mono.%	4.000 ± 0.6000	5.700 ± 1.642	0.2828
Eosin.%	2.500 ± 0.2000	4.940 ± 0.9553	0.0941
RBC	7.360 ± 1.060	7.156 ± 0.5533	0.4290
HGB	12.20 ± 1.500	12.38 ± 1.048	0.4646
HCT	36.05 ± 4.050	37.82 ± 3.269	0.3877
MCV	49.20 ± 1.600	52.76 ± 0.9405	0.0515
MCH	16.65 ± 0.3500	17.28 ± 0.2354	0.1037
MCHC	33.80 ± 0.4000	32.76 ± 0.3696	0.0862
RDW-CV	17.05 ± 3.150	15.74 ± 1.105	0.3106
RDW-SD	27.00 ± 2.000	27.70 ± 1.064	0.3746
PLT	696.5 ± 324.5	498.4 ± 166.3	0.2855

## Abbreviations

ED-XRF: Energy dispersive X-ray
RDA: Recommended daily allowance
UNCP: Unsweetened natural cocoa powder
ALT: Alanine aminotransferase
AST: Aspartate aminotransferase
CK: Creatinine kinase
ALB: Albumin
ALP: Alkaline phosphatase
HDL: High density lipoprotein
LDL: Low density lipoprotein
VLDL: Very low density lipoproteins
ANOVA: Analysis of variance
GAFCO: Ghana Agriculture Food Company
LD_50_: Lethal dose
SD: Sprague-Dawley
SDR: Sprague-Dawley Rats
HCT: Haematocrit
HD: High dose
HGB: Haemoglobin
LD: Low dose
LYM%: Lymphocytes percentage
LYM#: Lymphocyte count
MCH: Mean corpuscular haemoglobin
MCHC: Mean corpuscular haemoglobin concentration
MCV: Mean corpuscular volume
MPV: Mean platelet volume
PDW: Platelet distribution width
P-LCR: Platelet larger cell ratio
PLT: Platelet
RBC: Red blood cells
RDW-CV: Coefficient of variation in red cell distribution width
RDW-SD: Standard deviation in red cell distribution width
TP: Total protein
WBC: White blood cells
Lympho.: Lymphocytes
Eosin.: Eosinophils
Baso.: Basophils
RDW-SD: Standard deviation in red cell distribution width
RDW-CV: Coefficient of variation in red cell distribution width
PDW: Platelet distribution width
MPV: Mean platelet volume.
